# Trusted Data Analysis and Consensus Mechanism of Product Traceability Based on Blockchain

**DOI:** 10.1155/2022/3035231

**Published:** 2022-08-27

**Authors:** Yang Kang, Qiang Li, Yuanyong Liu

**Affiliations:** College of Artificial Intelligence, Chongqing University of Arts and Sciences, Yongchuan 402160, Chongqing, China

## Abstract

As a decentralized, distributed system between functional and benefit management functions, blockchain is effective for financial transaction data security, data tracking and antitampering, product tracking, and access control. In this context, we have conducted experimental research on the blockchain product traceability trusted data analysis consensus mechanism and reached the following conclusions. (1) There are decentralization, irreversible tampering, traceability, and openness through the blockchain's own information and other functions so that a series of processes from raw material production, transportation, and logistics sales are well documented. (2) Under the same network environment, if the number of matches in the system increases, the average matching time consumed by the original engine is greater than the average matching time on the optimized engine. For example, taking 10% of Byzantine nodes in the system, the number of consensus increases, the average ITPBFT consensus time is about 5.74 s, and the average consensus time of the PBFT consensus mechanism is about 6.13 s. As a decentralized distributed data management system through nodes, blockchain is widely used in financial transactions, copyright protection, and product areas such as tracking and access control. In this regard, we conducted an experimental study of the consensus mechanism to analyze reliable data on the traceability of blockchain products and came to the conclusion of the experiment.

## 1. Introduction

Driven by the recent surge in interest in blockchains, it was investigated whether they would be ideal for the Internet of Things (IoT). Blockchain supports networks where untrusted participants communicate together in a verifiable manner without a trusted broker. We study the operation of this mechanism and evaluate smart contract scenarios which are in the blockchain and allow you to automate multistage processes. Then, we move into the IoT field and describe how the combination of blockchain and IoT can facilitate sharing of services and resources and create a market for interoperable services [1]. The performance of a probabilistic proof-of-work- (PoW-) based consensus structure, also known as a blockchain, is not a major concern. It is a success story despite its consensus latency of around hours, and the theoretical maximum throughput is only 7 transactions per second. The current situation is very different and the poor performance scalability of the initial PoW blocks no longer makes sense. In particular, platform trends support the implementation of randomly distributed applications across block structures, and the need for better performance has entered the realm of database replication protocols [2]. Blockchain is a distributed transaction and information management technology first developed for the Bitcoin cryptocurrency. Interest in blockchain technology has grown since the idea was proposed in 2008. Interest in blockchain lies in the fact that its key features ensure security, anonymity, and data integrity without third-party control. This makes it an interesting area of research, especially in terms of technical issues and limitations [3]. Block chains are a new cryptographic and IT application to solve an old financial reporting problem and can lead to extensive changes in corporate governance. Many major financial players have already started investing in the new technology, with exchanges proposing the use of block chain as a new way to trade in company shares and track ownership. The effects of these changes on directors, institutional investors, minority shareholders, auditors, and other parts of the corporate governance system are reviewed. The lower costs, higher liquidity, more detailed information, and ownership transparency offered by blockchain can significantly improve the balance of power between these groups [4]. The main advantages of blockchain are decentralization, time series data, shared services, programmability, and security, making it particularly suitable for building programmable monetary systems, financial systems, and even macrosocial systems. The basic model of the blockchain system is proposed, and the principles, technologies, methods, and applications of the blockchain and related Bitcoin systems are discussed. We also discuss smart contracts and their applications and show future trends in a blockchain parallel society. It provides useful guidance and reference for future research work [5]. At present, several Chinese departments have invested a lot of money and effort in setting up proper food control systems. As a research team that has developed and applied traceability systems for several years, this article presents methods to achieve effective traceability and highlights the importance of traceability systems. The scientific elements of the proposed traceability system will be used to explore the usefulness of analytical data and the development of a food safety assessment system. The above methods can be used to produce useful and effective information to improve corporate governance [6]. Logistics helps companies optimize the entire enterprise supply chain from one source to another to achieve their strategic goals. Traceability has become one of the integrated components of supply chain management, supply chain monitoring, and data monitoring at various points at the beginning and end of a product. Depending on their role, all participants in the supply chain have different goals for implementing monitoring solutions [7]. Blockchain technology is a distributed ledger that allows you to transfer data seamlessly, securely, and reliably. The benefits of introducing blockchain technology to manage and control PSA products in the supply chain include decentralized management, security, tracking capabilities, and verified operational planning [8]. Elaborated through the required performance analysis with data traceability. The implementation of the proposed EWRDN service for sexual functionality is the main contribution of this work. A trusted authoritative service coordination is also introduced to operate the proposed service. The results show that the proposed service synergy can provide the traceability function of data and experimentally defends against most experienced data security threats. Additionally, the EWRDN service has been shown to provide two different data compression qualities as a differentiated quality service. It has been shown that in EWRDN coordination, a fixed compression recovery rate has been achieved [9]. There is a traceability link between each new requirement and the objects predicted to change in the design object model. When asked, they claimed they were confident that the correct number of objects would be changed, but they were less sure what was needed number of hours worked. Analysis of impact analysis shows that predictions for individual objects are often nearly correct, but the number of objects actually changed is often larger than the number of objects predicted [10]. The multiterminal controllable security intelligent system not only adopts the proprietary protocol to fight against wireless network intrusion and system vulnerability mining program to realize the secure encrypted transmission of multiterminal data exchange but also establishes a consensus environment and uses the consensus mechanism to record and verify the security status of the access device to ensure the security of the system. The system reduces the cost of establishing a security architecture and the requirements for computing power and can better cope with the frequent occurrence of security incidents [11]. The current architecture of the risk scoring system is highly centralized, which can lead to issues such as data loss, bias, and decentralization. Along with blockchain technology, it has the features of decentralization, security and reliability, integrated service, and consistency. A new architecture of a blockchain-based risk assessment system and an enhanced DPOS-based consensus mechanism algorithm is proposed [12]. Strengthening the management and support of nonconsensus basic research is conducive to promoting the original innovation of basic research, and local science and technology departments are increasingly interested in exploring the management mechanism of nonconsensus basic research. Combined with management experience, policy recommendations are put forward from two aspects: the establishment of nonconsensus basic research special funds and the design of nonconsensus project selection mechanism, to provide certain theoretical guidance for the management practice of nonconsensus basic research in the future [13]. The blockchain and D-IoT equivalent architectures are based on a master-slave multichain. The integrated equivalent architecture provides key applications of blockchain technology as a unified mechanism and smart contract in D-IoT. The broad perspective for blockchain deployment in D-IoT is explored [14]. There are several general consensus algorithms in blockchain technology. They differ in the complexity of calculating fault tolerance and fault tolerance. The implementation, consistency, scalability, and efficiency of blockchain consensus mechanisms require further refinement and improvement. Mechanisms for code approval and implementation of Bitcoin, Ethereum, and Hyperledger are reviewed, discussed, and proposed in [15].

## 2. Trusted Data Analysis of Product Traceability of Blockchain

### 2.1. Blockchain Technology

1. Decentralization: the blockchain uses peer-to-peer technology to store data, using distributed accounting and storage. All nodes have equal rights and obligations
2. Security: information security cannot be tampered with
3. History: any transaction records will be stored in the database
4. Collective maintenance: in an open system, private information in transactions is encrypted, and all nodes with maintenance functions are jointly maintained. Anyone can query each blockchain data through an open interface, so the entire system information is highly transparent

Blockchain technology is considered the key technology most likely to power the fifth wave of disruption. It is a distributed ledger technology consisting of distributed databases, encryption algorithms, consensus mechanisms, and smart protocols. Based on the characteristics of openness, transparency, decentralization, and nontampering of blockchain data, it has received more and more attention, and it has gradually become materialized from the concept, from the initial application of financial currency to applications in various fields.

*Encryption Algorithm*. The encryption algorithm is the cornerstone of the blockchain. Blockchain uses hash algorithms and asymmetric encryption technology to ensure the irreversibility and security of blockchain technology. Hash algorithms, also known as secure hash algorithms, are widely used in many encryption technologies, including blockchain technology. It maps data of any length to a fixed-length hash queue according to certain rules. Algorithms, the most common being the SHA-1, SHA-2, and SHA-3 families, MD5, etc.

*Consensus Mechanism*. The consensus mechanism is the key blockchain mechanism. The blockchain consensus mechanism introduces Basic Certificate (PoW), Proof of Contribution (POS), Delegated Proof of Contribution (DPOS), Practical Byzantine Error Resistance (PBFT), etc. to adapt to the consensus system of each platform. The consensus mechanism is used to solve the consensus problem of distributed systems. It first appeared in 1975, and the blockchain consensus mechanism was the proof-of-work mechanism proposed by Satoshi Nakamoto in Bitcoin core.

**Figure 1:**
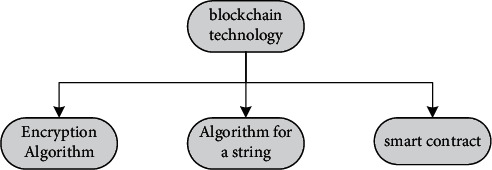
Blockchain technology.

*Smart Contract*. A smart contract is essentially a computer protocol that allows the blockchain application platform to operate automatically and unconditionally according to the proposed contract terms, thereby replacing the uncertainty of manual calculations and operations used in traditional business practices to perform contracts. It greatly improves the interaction and circulation of information nodes. Smart contracts have a certain ability for independent judgment and self-execution. Because they are based on programming languages, they do not need to rely on third parties or centralized institutions and cannot be intervened by humans, so they have high efficiency and accurate and strong execution capabilities. The technical types of blockchain are shown in Figure 1.

### 2.2. Blockchain Product Traceability

**Figure 2:**
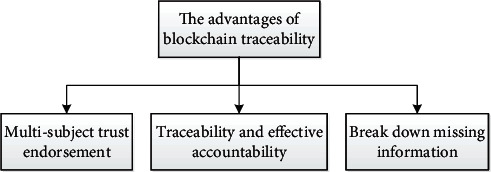
The advantages of blockchain traceability.

With the continuous improvement of material living standards, product updates and iterations have become more and more frequent, resulting in the emergence of many electronic foundry companies, small workshops, etc., which have threatened the safety of electronic use. The trust mechanism between consumers and consumers has not been perfected. The main raw materials, parts, production, processing, assembly, supply, and all aspects of logistics involved in the production of products will involve many supply chains, distributors, software and hardware companies, and so on. The traceability of the product cannot be fully maintained and guaranteed at all. A series of processes that make products from raw material production, transportation, and logistics sales are well documented. Compared with other traditional traceability methods, the advantages of blockchain traceability can be roughly summarized into the following three basic aspects:

1. Multiparty confirmation: use blockchain features such as decentralization, irreversibility, and traceability to support product trust, encourage more participants in the supply chain to jointly collect and maintain product information, and increase system trust.
2. Traceability and accountability: the blockchain adopts distributed ledger technology to ensure that data records cannot be tampered with, making commodity information and transaction information transparent, authentic, and traceable. Consumers can view product information and eliminate the problem of fake and inferior products. Enterprises can effectively and quickly trace the cause and source of electronic product problems and quickly recall all problematic products to reduce financial losses.
3. Hacker data warehouse of all parties: when these data are packaged and stored in blocks, supply chain participants jointly maintain their own data sources, which can realize information disclosure. All parties can query the process information through the system to ensure that all participants find out the problems in their operation in time, which helps to improve the management efficiency of the entire supply chain. The advantages of blockchain product traceability are shown in Figure 2.

### 2.3. Product Traceability Method

**Figure 3:**
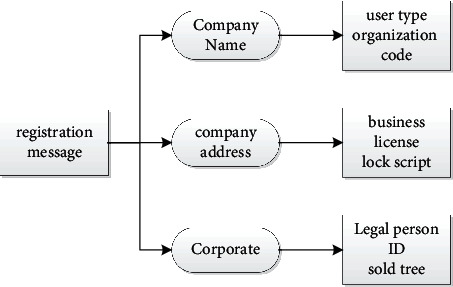
Registration information data structure.

The blockchain approach to product tracking involves each merchant and consumer registering an address before using the system. More event information for this address is as follows. When selling an item, the seller must provide the system with information about the transaction. When consumers buy a product, they can verify the product through the QR code in the system before purchasing. The first level of the system is Consumer, Outstanding Supplier, and Production Log in to create a public/private key pair and use the revocation script and personal information to register and authenticate to the system, as shown in Figure 3 to add information to the registration: company name, address, corporate legal entity, etc. The registration node sends registration information, and other nodes cache the registration information after receiving the message, making a smart trade. Registration information is collected in blocks awaiting approval if the message conforms to all terms of the smart agreement. If the message does not meet all the requirements of the smart contract, the message is removed from the cache. Review intelligence logs to ensure that information provided by controllers is the property of individuals and to prevent attackers from maliciously recording information about others. Check the contents of the “Authentication Information” field of the registration information. Registration information performs three functions: first, to inform consumers of the legitimacy of transactions, second, to trace incidents involving commodities, and third, to prevent manufacturers from releasing counterfeit products. It returns success if all conditions are true and returns an error if any test fails.

## 3. Blockchain Algorithms

### 3.1. Adaptive PoW Algorithm

1. The node monitors the data records of the entire network, and the data records that pass the basic legality verification will be temporarily stored.
2. After finding a reasonable random number, generate block information, first enter the block header information, and then the data record information.
3. The newly generated block is broadcasted to the outside after receiving the order. After other nodes have passed the verification, they will be connected to the blockchain, and the height of the main chain will be increased by one. Then, all nodes will switch to the new block and continue to perform workload proof and block. Production.

To improve the efficiency of end node transactions on the local network, end nodes use a handshake protocol called an adaptive handshake algorithm. The algorithm dynamically adjusts the complexity of implementing the PoW algorithm according to the behavior of end nodes. At the same time, the security risk in the local network is reduced by reducing the complexity of the PoW algorithm. The correct operation of the A-PoW algorithm depends on the results of the local network node edge verification mechanism checks. The following describes how the node bounds checking mechanism works. We describe how edge nodes calculate the contribution of the corresponding node based on the behavior of the node under test. We create nodes in a variety of ways and ultimately ensure that the node's input value calculation formula effectively and accurately represents the node's contribution to the input blockchain. We calculate node contribution using the following formula:

(1) $$\begin{array}{c} C_{i}=\lambda_{1}C_{i}^{p}+\lambda_{2}C_{i}^{N}. \end{array}$$

To study the influence of different types of transactions on the contribution amount, *C*_*i*_^*N*^ represents the influence of malicious behavior on the contribution value, *λ*_1_ and *λ*_2_ represent the weight of these two effects, and the formula can be expressed as

(2) $$\begin{array}{c} C_{i}^{p}=\sum_{k=1}^{n}T_{1}\left(t\right)*a\left(b\right). \end{array}$$

Among them, *n* is the number of events published by the entire node in unit time, *T*1(*t*) is a function of time damping, its value gradually decreases with time, and when the importance is greater, *T*1(*t*) can be expressed as

(3) $$\begin{array}{c} T_{1}\left(t\right)=N_{0}e^{-a\left(t+1\right)}, \end{array}$$

(4) $$\begin{array}{c} a=\frac{1}{m}\ln\left(\frac{N_{\text{INIT}}}{N_{\text{FINISH}}}\right), \end{array}$$

(5) $$\begin{array}{c} l=\frac{1}{a}\ln\left(\frac{N_{0}}{N_{\text{init}}}\right), \end{array}$$

*N*0 in formula (3) is the initial value of the function, *a* is called an exponential decay variable, and *l* is the inversion to the left, so the value of *T*1(*t*) can be calculated from any position instead of *N*0. According to equations (4) and (5), the decay function of *N*_init_ begins to decrease and decreases to *N*_finish_ after *m* unit time. *A*(*b*) represents the actual impact on the payment amount. When the host requests authentication, if there are pending events, *a*(*b*) has the value *N*; otherwise, *a*(*b*) has the value −*N*. Therefore, the payment calculation formula encourages nodes to actively look at these “pending” events. The impact factor of the detected harmful payment behavior can be expressed as

(6) $$\begin{array}{c} C_{I}^{N}=\sum_{k=1}^{n}T_{2}\left(i\right)*\beta \left(b\right). \end{array}$$

The system assumes relatively no malicious activity. Harmful activity detected during this check affects not only the number of attachments calculated at this time but also the number of attachments in the future. As shown in the formula, *T*_2_(*i*) is a function of temporary damping, which can be written as

(7) $$\begin{array}{c} T_{2}\left(i\right)=0.8^{i}, \end{array}$$

where Β(*b*) in the *C*_*I*_^*N*^ calculation formula represents the degree of damage caused by malicious behavior. Assuming that in the current system, the threshold for publishing transactions through terminal nodes is *M*, and the number of transactions performed during this analysis *T* period is *N*, then *β*(*b*) can be expressed as

(8) $$\begin{array}{c} \beta \left(b\right)=\left\{\begin{array}{cc} 0, & N<M,\\ K*\left(N-M\right), & N<M. \end{array}\right. \end{array}$$

If the number of errors is below the threshold, there is no penalty. If the trading volume exceeds 10%, the value of *k* is relatively large. If the additional transaction volume does not exceed 10%, the edge node considers this to be an acceptable margin of error. Therefore, the value of *k* is relatively small. Meanwhile, *T*2(*t*) is a function that is repeatedly decomposed in control events. Formula (1) is the number of breakpoints between the malicious activity's breakpoint and the current breakpoint.

### 3.2. Affinity Propagation Clustering Algorithm

Since the number of game participants and the number of solutions have a great influence on the complexity of the game, in order to solve this problem, this paper firstly organizes the data and regards the clustering of the game as a category of participants, which not only simplifies the game process. At the same time, the original structure and function of the data are preserved. If participants rely on clusters, they will not be penalized if the number falls below a threshold. When the trading volume exceeds 10%, the value of *k* is relatively large. If the number of transactions exceeds 10%, the edge node is considered to have an error range. So, the value of *k* is relatively small. Meanwhile, *T*2(*t*) is a function that is repeatedly decomposed in checkpoint events. The *i* formula represents the breakpoint between the malicious behavior and the current breakpoint (the number of breakpoints between intervals). That is, the longer the malicious behavior is, the lower the *T*2(*t*) is. The result is to observe the data characteristics of each cluster and further analyze a specific set of clusters to make decisions. The silhouette factor provides a nice scoring function to determine the best score. The silhouette coefficient is one of the commonly used performance metrics to evaluate cluster cohesion and separation, defined as follows:

(9) $$\begin{array}{c} s\left(i\right)=\frac{b\left(i\right)-a\left(i\right)}{\text{max}\left\{b\left(i\right)-a\left(i\right)\right\}}. \end{array}$$

The higher the silhouette factor, the better the clustering effect. The quality of the grouping is evaluated by silhouette coefficients corresponding to different reference values. We choose a Nash equilibrium solution and find the best path. This paper captures the best idea of finding the optimal solution, uses the advanced particle swarm algorithm to achieve the optimal global resource allocation, and develops and solves the Nash equilibrium solution for various industries. The position of each particle in the algorithm represents the situation of an investment company. Using this algorithm, we display the origin of all particles in [0, 1] and determine the origin of the particle.

(10) $$\begin{array}{c} N_{X,Y}=\frac{N_{\text{max}}-N_{\text{min}}}{O_{\text{max}}-O_{\text{min}}}\times \left(O_{x,y}-O_{\text{min}}\right)+N_{\text{min}}. \end{array}$$

The optimal idea is used to find the optimal solution, the improved particle clustering algorithm is used to obtain the overall optimal distribution of resources, and the Nash equilibrium solution is designed and opened for each type of industry. The position of each particle in the algorithm represents the situation in which the investment company operates. The starting positions of all particles in the algorithm are plotted on [0, 1], and the initial positions of the particles are determined.

In the game situation, the strategy selected by the player optimally matches the strategies of other players. In the game scenario, the strategy selected by the player corresponds well with the strategies of other players. Therefore, the particle in the optimal state is the Nash equilibrium in the game, according to the definition and properties of *N*_ash_ equilibrium. The game strategic planning based on *n*-person limited refusal to cooperate is as follows:

(11) $$\begin{array}{c} u_{i}=\text{max}\left\{{\text{sreg}}_{i}-\left(E\left(x\right)-E_{\text{avg}}\right),0\right\}. \end{array}$$

If the mixed situation according to equation (11) is a Nash equilibrium solution, *u*_*i*_ can obtain the minimum value of 0, and when the mixed situation is a non-Nash equilibrium solution, *u*_*i*_ can obtain a larger value. In order to evaluate the Nash equilibrium solution and obtain an approximate Nash equilibrium solution, this article will develop the fitness function in the investment board game scenario:

(12) $$\begin{array}{c} f\left(x\right)=\min u_{i},\;i\in \left(1,2,\ldots ,n\right). \end{array}$$

In the game strategy combination space, the smaller the condition of *f*(*x*) is, the closer the enterprise profit is to the equilibrium value. The compaction rate method selects the global optimal position and guides the particles to search the sparse area, so that it does not fall into the local optimum, improves the understanding of variability, and finally adheres to the game balance for adaptation. This method takes time. As the data accumulates or the complexity increases on a large scale, the performance of the algorithm degrades. In intelligent algorithms, the data processing capacity is limited and the accuracy is insufficient. In this paper, the gravity search algorithm is used to improve the particle flow algorithm to improve the overall optimization ability. GSA is a new heuristic method. The motion of particles follows the laws of dynamics. Particles move toward particles with higher mass, and particles with higher mass are in the best position to obtain the optimal solution to the problem. Learning the value of the particle state function determines each particle's mass and force, calculates acceleration, and updates velocity and position. The steps to calculate these parameters are as follows:

(13) $$\begin{array}{c} m_{i}\left(t\right)=\frac{{\text{fit}}_{i}\left(t\right)-\text{worst}\left(t\right)}{\text{best}\left(t\right)-\text{worst}\left(t\right)},\\ M_{i}\left(t\right)=\frac{m_{i}\left(t\right)}{\sum_{I=1}^{N}m_{j}\left(t\right)}. \end{array}$$

Calculate gravity. The gravitational force of particle *j* on particle *i* is

(14) $$\begin{array}{c} F_{ij}\left(t\right)=G\left(t\right)\frac{M_{pi}\left(t\right)\times M_{aj}}{R_{ij}\left(t\right)+\varepsilon }\left(x_{j}\left(t\right)-x_{i}\left(t\right)\right). \end{array}$$

The gravity detection algorithm is used to improve the particle swarm algorithm to improve global optimization capabilities. GSA is a new research method. The movement of particles obeys the laws of kinetics. Particles move in the direction of the particles with the greatest force, and the most powerful particles are in the best position to obtain the best solution to the problem. By evaluating the value of the particle's fitness function, the force and strength of each particle are determined, the acceleration is calculated, and the speed and position are updated.

In the formula, *G*(*t*) represents the value of the gravitational constant, *M*_*pi*_(*t*) represents the inertial mass of the acted particle *i*, *M*_*aj*_ represents the inertial mass of the acting particle *j*, and *ε* is a small constant to avoid the denominator being 0. The net force is then applied to part *i*.

(15) $$\begin{array}{c} F_{I}\left(t\right)=\sum_{J=1}^{N}\text{rand}\times F_{ij}\left(t\right), \end{array}$$

where rand is the reference value for calculating acceleration. According to the law, the acceleration of particle *i* is

(16) $$\begin{array}{c} a_{i}\left(t\right)=\frac{F_{I}\left(t\right)}{M_{i}\left(t\right)}. \end{array}$$

In this paper, the particle swarm algorithm is used to solve the Nash equilibrium solution, and the clustering distance calculation method is used to control the particle search process.

### 3.3. Homomorphic Encryption Algorithm

Encryption algorithms can achieve additive homomorphic properties, which can meet the needs of many privacy-preserving applications. In particular, the Payet encryption algorithm can be composed of three parts, namely, key generation, data encryption, and data encryption. Generate the key: according to the specified security parameter *k*, first select two sufficiently large prime numbers, where the sum of these two prime numbers is *k*, and then calculate the RSA modulus *N*. Define a function.

(17) $$\begin{array}{c} L\left(\mu \right)=\frac{\mu -1}{N}. \end{array}$$

Then, choose a suitable generator, and use the above parameters to calculate. Finally, the formula in the Payet encryption algorithm is obtained. *p*_*k*_ = (*N*, *g*), and its corresponding formula *m* ∈ *Z*_*N*_. Data encryption: according to the given message *m* ∈ *Z*_*N*_, choose a random number *r* ∈ *Z*_*N*_^*∗*^, so the file can be calculated by the following formula:

(18) $$\begin{array}{c} c=E\left(M\right)=g^{m}.r^{n}\text{mod}N^{2}. \end{array}$$

Data decryption: according to the ciphertext *C* ∈ *Z*_*N*_^*∗*^ calculated above, the corresponding ciphertext *m* before encryption can be calculated by the following formula:

(19) $$\begin{array}{c} m=D\left(C\right)=L\left(C^{\text{mod}N^{2}}\right). \end{array}$$

It should be noted that the Payet encryption algorithm is semantically secure and can resist multiple plaintext attacks, and its accuracy and security can be verified.

## 4. Research on Consensus Mechanism of Product Traceability Trusted Data Analysis of Blockchain

### 4.1. Analysis of Product Traceability under Different Blockchain Algorithms

This paper examines the time between transaction validation and block generation and compares the PoVT proposed in CDBFT to the PBFT used in Hyperledger Fabric. The initial total number of nodes in the system is fixed at 10, and the number of transfers per block is 1 to 500. It is shown in Table 1.

**Figure 4:**
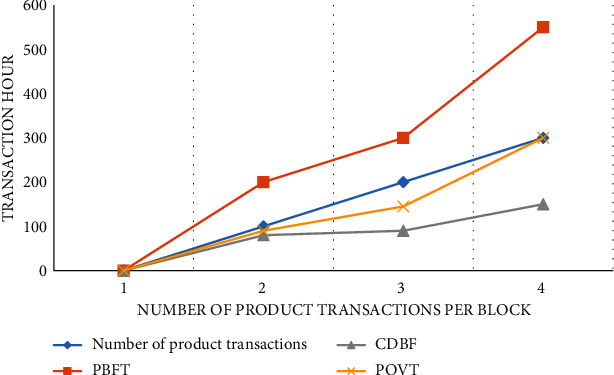
Time changes of PoVT algorithm when the node is fixed.

In Figure 4, the required time is shown as a function of the number of events in the block. The comparison shows that for multiple simultaneous 100 events, PBFT lasts 200 ms, CDBFT lasts 120 ms, and PoVT lasts 80 ms. Correspondingly, for 30 events, the PBFT gain time is 600 ms, the CDBFT is 350 ms, and the PoVT is about 210 ms. As the number of simultaneous events increases, so does the confirmation time for all three. However, the computation time oT is higher than PBT and CDBFT.

**Figure 5:**
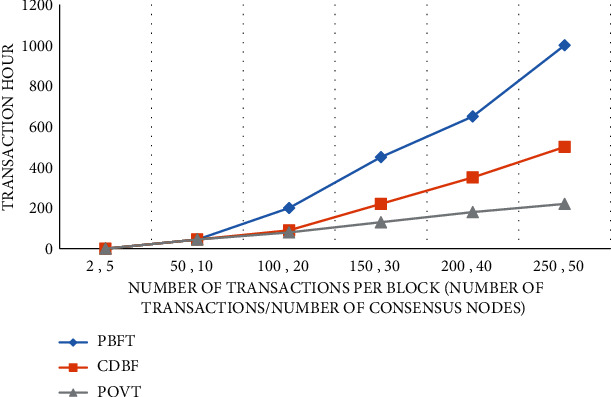
Time changes of PoVT algorithm when the number of nodes and transactions is variable.

Figure 5 compares the time required to complete a transaction under two different scenario changes. The horizontal axis represents the number of events in each block and the number of participants in the corresponding consensus. In the consensus algorithm proposed in this paper, each session has 20 consensus nodes and 100 events. PBFT acceptance time increases significantly (200 ms), CDBFT takes 100 ms, and PoVT takes only 80 ms. From the analysis point of view, PoVT takes less time to complete the transaction. PBFT reaches consensus through a full three-step broadcast, while the CDBFT system takes a long time to calculate voting fees, fines, and the credit evaluation process. If 100% of the nodes participate in the consensus process, the time required will gradually increase. The solution proposed in this paper is to achieve consensus by selecting multiple nodes and reducing the amount of communication, which can effectively reduce the communication cost.

**Figure 6:**
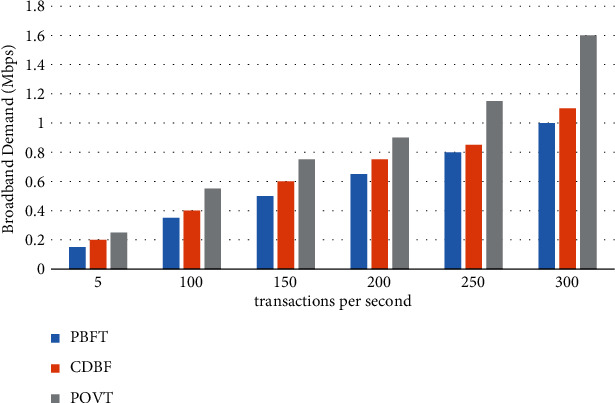
Changes in bandwidth demand as the number of concurrent transactions increases.

In Figure 6, we see the importance of ensuring the performance required to achieve optimal transaction rates, as consensus must be formed when a particular block is delivered. Bandwidth requirements are measured by varying the number of simultaneous events per second and the number of participating nodes. Bandwidth requirements increase with the number of simultaneous events, not directly with the number of IoT nodes. In traditional blockchains, this demand is quasi-linear and proportional to the increase in transaction volume.

### 4.2. Product Traceability Trusted Data Analysis

**Figure 7:**
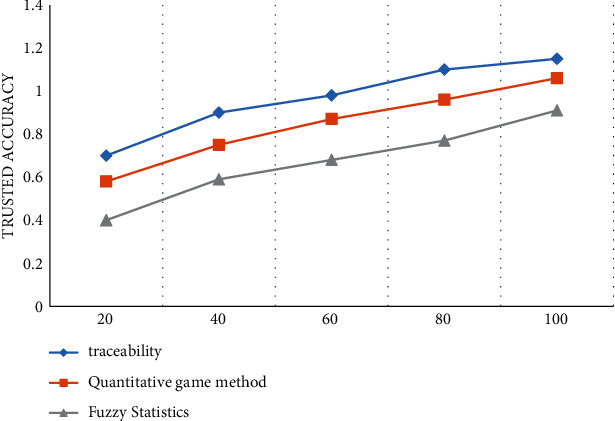
Credibility comparison test.

We can conclude that the method in this article analyzes the reliable data of product traceability, and the accuracy of the effect evaluation is high. The experiment also conducts a reliability test of the method presented in the article and compares it with the statistically ambiguous method and the digital game method. The results are shown in Figure 7.

From the experimental data in Figure 7, we can know that the reliability of reliable traceability tracking is highest among the three methods. The number of iterations can be up to 50, the confidence level can be up to 1, and the number of iterations of obscure statistical methods with quantitative estimates can be up to 80. Up to 90 repetitions of game mods, reliability can reach 1.

### 4.3. Research on Consensus Mechanism of Blockchain Product Traceability

The security of blockchain consensus protocols is often related to feasibility. The security evaluation of the consensus protocol proposed in this paper is carried out in terms of durability and performance.

**Figure 8:**
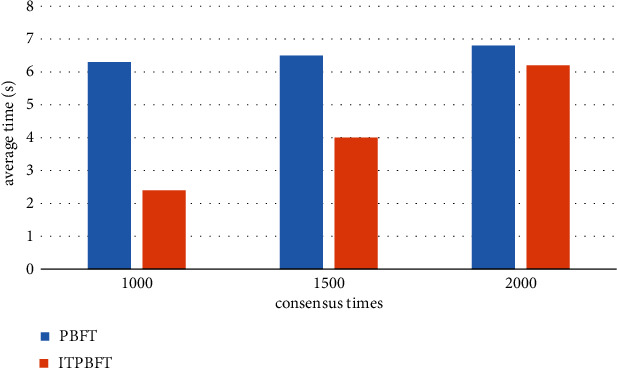
Average consensus time when the number of consensus increases.

**Figure 9:**
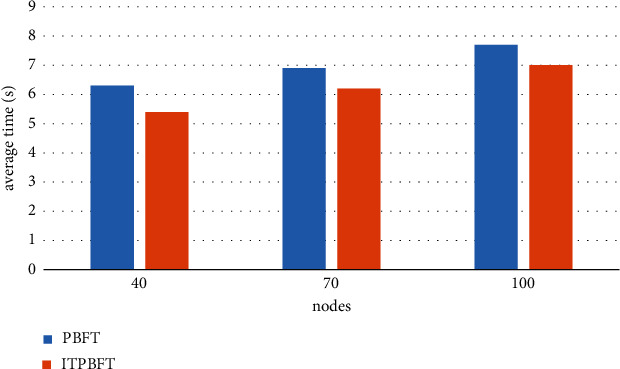
Average consensus time when nodes are incremented.

It can be seen from Table 2 that the selection times of each node are not related to each other, the maximum error rate can be allowed in the design, and the selection times of each node are roughly the same, which are all possible events. That is, all nodes are selected approximately the same number of times in each cycle. Also, the maximum number of selection errors does not depend on the total number of nodes. In terms of consensus effectiveness, the experiment mainly estimates and evaluates the average consensus time of these two mechanisms, and the average consensus time is shorter and the consensus efficiency is higher. The test graph environment selects 10%, 20%, and 30% of Byzantine nodes in the network. The number of system nodes *n* is 52, the number of hits is increased to 2000, and the corresponding number *t* is 1500. Under certain conditions, the number of system nodes increases to 100. We compare the consensus performance of the two mechanisms under different conditions, check the consensus number, the number of nodes, and other factors, repeat the experiment 100 times, and run the average test results. The results are shown in Figures 8 and 9.

In Tables 3 and 4, the following are experimental results for network environments with 10%, 20%, and 30% Byzantine nodes. According to the test results, the average consensus time of the main mechanism under the same network environment is higher than the average consensus time of the optimized mechanism in the system. For example, the number of Byzantine nodes in the system has increased by 10%, and the average repair time of ITPBFT is about 5.74 seconds. The average fitting time of the PBFT consensus engine is about 6.13 seconds; in addition, as the number of nodes in the system increases, the average consensus time used by the original engine is also higher than that of the optimized engine. The consensus efficiency of the optimized engine has been significantly improved compared to the original engine. For example, by using 10% of Byzantine nodes in the system, the optimization engine can increase consensus efficiency by about 4.7%. With the gradual growth of Byzantine Online, the improvement of consensus performance has become more and more obvious. There are 30% Byzantine nodes in the system. By increasing the consensus, the optimization engine can improve the consensus efficiency by about 18.5%; as the number of nodes increases, the optimization engine can improve by nearly 16% compared to the original engine. The test results show that although the approval efficiency of the two mechanisms decreases with the increase of the number of approvals and the number of nodes, the optimization engine can improve the approval efficiency and reduce the approval time.

## 5. Conclusion

As a distributed information system supported by nodes, blockchain is widely used in the fields of transaction, copyright protection, product control, and access control. The emergence of platforms such as Ethereum and Hyperledger has promoted the rapid development of blockchain technology. Although blockchain technology is popular, high-power consumption and poor performance also affect the development of blockchain. The consensus mechanism is the core of blockchain technology: it is the basic mechanism for nodes to sequence events during model implementation, ensuring that nodes reach a consistent and secure network during delivery.

## Figures and Tables

**Table 1:** Number of block products under different algorithms.

Number of product transactions	0	100	200	300	400	500
PBFT	0	200	300	550	800	1000
CDBF	0	80	90	150	280	390
PoVT	0	90	145	300	400	550

**Table 2:** Statistical table of data related to node election in four cases.

Number of elections	Trend line function	Maximum number of errors	Maximum error rate (%)	R.R
10000	*Y* = 400	13	3.35	0.0078
10000	*Y* = 200	11	5.50	0.0015
10000	*Y* = 133.3	9.3	6.97	0.0026
10000	*Y* = 100	6	6	0.0017

**Table 3:** Average consensus schedule with increasing consensus times.

Mechanism	1000	1500	2000
PBFT	6.3	6.5	6.8
ITPBFT	2.4	4	6.2

**Table 4:** Average consensus time when nodes are incremented.

Mechanism	40	70	100
PBFT	6.3	6.9	7.7
ITPBFT	5.4	6.2	7

## Data Availability

The experimental data used to support the findings of this study are available from the corresponding author upon request.
